# Machine learning methods for predicting essential metabolic genes from *Plasmodium falciparum* genome-scale metabolic network

**DOI:** 10.1371/journal.pone.0315530

**Published:** 2024-12-23

**Authors:** Itunuoluwa Isewon, Stephen Binaansim, Faith Adegoke, Jerry Emmanuel, Jelili Oyelade

**Affiliations:** 1 Department of Computer and Information Sciences, Covenant University, Ota, Ogun State, Nigeria; 2 Covenant Bioinformatics Research (CUBRe), Covenant University, Ota, Nigeria; 3 Covenant Applied Informatics and Communication, Africa Centre of Excellence (CApIC-ACE), Covenant University, Ota, Ogun State, Nigeria; Federal University Dutse, NIGERIA

## Abstract

Essential genes are those whose presence is vital for a cell’s survival and growth. Detecting these genes in disease-causing organisms is critical for various biological studies, including understanding microbe metabolism, engineering genetically modified microorganisms, and identifying targets for treatment. When essential genes are expressed, they give rise to essential proteins. Identifying these genes, especially in complex organisms like *Plasmodium falciparum*, which causes malaria, is challenging due to the cost and time associated with experimental methods. Thus, computational approaches have emerged. Early research in this area prioritised the study of less intricate organisms, inadvertently neglecting the complexities of metabolite transport in metabolic networks. To overcome this, a Network-based Machine Learning framework was proposed. It assessed various network properties in *Plasmodium falciparum*, using a Genome-Scale Metabolic Model (iAM_Pf480) from the BiGG database and essentiality data from the Ogee database. The proposed approach substantially improved gene essentiality predictions as it considered the weighted and directed nature of metabolic networks and utilised network-based features, achieving a high accuracy rate of 0.85 and an AuROC of 0.7. Furthermore, this study enhanced the understanding of metabolic networks and their role in determining gene essentiality in *Plasmodium falciparum*. Notably, our model identified 9 genes previously considered non-essential in the Ogee database but now predicted to be essential, with some of them potentially serving as drug targets for malaria treatment, thereby opening exciting research avenues.

## Introduction

Malaria remains a major global health concern, with *Plasmodium falciparum* being one of the deadliest human malaria parasites [1]. The emergence of drug-resistant strains and the limited success of current treatment strategies lay emphasis on the need for innovative approaches to combat this disease. Millions of malaria cases are caused by this parasitic eukaryotic organism, which has a disproportionately negative impact on low- and middle-income African nations [2]. The 2023 World Malaria Report highlights a concerning increase in malaria infections and fatalities. In 2022, there were an estimated 249 million malaria cases, with 608,000 deaths, which reflects a slight rise of 5 million cases above 2021 in the 2022 WHO report [3]. Accurate identification of *P*. *falciparum* essential genes is a vital step and bears promise as new therapeutic targets for successful antimalarial drug development and identifying possible vaccine candidates [4]. Understanding the essentiality of certain genes allows researchers to focus on crucial enzymatic activities and pathways. One promising avenue is the identification of essential metabolic genes within the P. falciparum genome-scale metabolic network [2,5,6].

**Fig 1:**
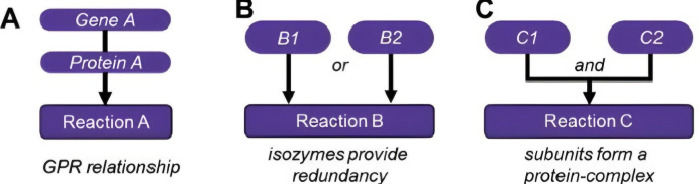
**Gene-protein-reaction (GPR) rules link genotype to phenotype** (A) GPR rule for an enzymatic reaction catalysed by a single gene’s protein product. (B) Redundant GPR rule where either protein B1 or B2 can independently catalyse the same function ("or" statement). (C) Complex GPR rule requiring both C1 and C2 for the reaction ("and" statement between non-redundant subunits) [8].

Metabolic essential genes are vital for cell survival. They encode enzymes for metabolic reactions crucial to systems biology [7]. The transcription and translation of these genes produce metabolic enzymes and proteins, which then catalyse metabolic reactions, based on the Gene-Protein Reaction (GPR) rule, which links genotype to phenotype as shown in Fig 1 [8].

The environment of a cell and the function of the gene determine whether it is vital for the survival of the cell [9,10]. Experimental methods, such as transposon mutagenesis, single gene deletion, antisense RNA, and Clustered Regularly Interspaced Short Palindromic Repeats (CRISPR) are often used to identify metabolic essential genes [11]. However, these experimental approaches are more expensive, intense, and time-consuming. Hence, computational techniques have been proposed, which is cost effective and can serve as a preliminary step to kick-start biological research into gene essentiality, and in recent years, this approach has become very popular [9,10,12].

Metabolic networks (MNs) are a type of biological network in which many concurrent chemical reactions and transport activities connect chemical molecules and other small chemical species known as metabolites [6]. Metabolic network modelling has made it possible to replicate various intracellular and intercellular processes to better understand how organisms work at the systemic level. The properties of the metabolic network at the structural, kinetic, and regulatory levels are inferred from the measurements of metabolite concentrations and reaction fluxes [13]. MNs contribute to the field of network medicine, playing a significant role in medical science research since they can be reconfigured to determine which pharmacological therapy-induced changes in network topography is harmful to the pathogen [14].

A genome-scale metabolic network/s (GSMN/s) are mathematical representations of metabolic networks that are developed from the context-specific annotated genome of a cell/organism [15]. A list of all biochemical processes and reactions in the cell, information on cellular boundaries, biomass reactions, and exchange reactions with the organism/cell environment to rebuild GSMNs, either manually or semi-manually [11,16]. To define realistic metabolic behaviour, the availability of nutrients and/or flux of other metabolites through a reaction per time are constrained, which provides insight into the state of the organism [17]. Flux Balance Analysis (FBA) is a mathematical optimization technique used in the study of metabolic models [18]. This has been observed in other applications in gene essentiality studies. Because of its breadth and applicability, FBA Modeling of metabolism is expanding dramatically, and its integration with omics data offers mechanistic insights into the genotype-phenotype environment relationship [19].

Although the application of FBA in gene essentiality studies in prokaryotes has produced encouraging results and led to more advanced gene essentiality research, FBA’s ability in pathogenic eukaryotes is substantially limited [20]. This is partly due to the limited quality of the available genome-scale metabolic models (GSMM/s) for eukaryotes that serve as imputes to FBA, and the fact that the prediction accuracy of FBA is quite sensitive to the biomass (i.e., objective function) that needs to be constantly adjusted to fit the environmental conditions under consideration. Growth rate maximisation is typically a biomass function (i.e., assuming that the cell will do all it can to maximise growth in any environmental condition). Additional objective functions, such as maximising Adenosine Triphosphate (ATP) production and lowering the substrate absorption rate, have also been proposed, it is still unclear whether this set objective works effectively across different species and/or under different environmental conditions [21]. It is also unknown whether deletion strains continue to try to optimise growth or whether gene deletions change cell physiology to achieve alternative survival aims that are not currently known [12]. Recently, there has been an increase in awareness of the considerable promise that integrating FBA with machine learning removes some of the core limitations of GSMN models and traditional FBA [13,22,23].

Machine learning (ML), a statistical technique that allows computers to "learn" internal systems from training data and to produce highly accurate predictions or classifications, has been used in GSMN research in recent years [18,24]. Numerous research studies and surveys have been conducted to determine whether ML techniques can be used in metabolic network research [25–27]. Graph theory has also emerged as an additional approach for gaining a deeper understanding of Metabolic Networks. In this method, these networks are represented as graph structures, and the features of these graphs are analysed to provide valuable biological insights into cell metabolism. Traditionally, metabolic networks are modelled as undirected bipartite graphs, where nodes represent both reactions and metabolites, and the graph is unweighted [28,29].

However, this modelling approach does not naturally capture the concept of flux distribution, which includes its flow and directionality, which is essential for understanding the flow of metabolites in the network. To address this limitation and provide a more comprehensive representation, Beguerisse-Díaz et al. introduced an innovative framework known as Mass Flow Graphs (MFG) in 2018 to construct flux-based graphs known as Mass Flow Graphs (MFGs) using organism-wide metabolic networks [30]. These graphs capture the direction of metabolic flows, with edges indicating the transfer of metabolites between source and target reactions. This approach enables the use of flux distributions from FBA, with or without specific biological contexts. When applied to Escherichia coli’s metabolic network, flux-dependent graphs revealed systematic topological and community structure changes under various environmental and genetic conditions. These changes give insights into metabolic flow rerouting and highlight the importance of key reactions and pathways, essential for understanding critical enzymatic processes [11,30].

In 2022, Freischem et al. adopted this approach and proposed a novel machine learning method to directly predict gene essentiality from wild-type flux distributions without assuming the optimality of deletion strains [11]. Their approach involved projecting the wild-type FBA solution onto a mass flow graph of E. coli and training binary classifiers on the connectivity features of graph nodes to predict gene essentiality. However, this approach has not yet been explored in pathogenic eukaryotic organisms. Additionally, the impact of other connectivity features on gene essentiality prediction has not yet been investigated.

This research study introduces a novel approach that integrates flux balance analysis, graph methods, and machine learning to predict essential metabolic reactions, and consequently the essential genes that codes them. In this approach we construct a metabolic network graph, weighted by flux, from iAM-Pf480, a *Plasmodium falciparum* genome-scale metabolic network (GSMM) adopted from the BiGG database [2]. The iAM-Pf480 dataset, spanning multiple genetic and biochemical aspects [1,6,15], is utilised to derive network-based features for predicting metabolic gene essentiality in *P*. *falciparum*. Our model demonstrates remarkable performance, uncovering previously non-essential genes. Some of these newly identified genes have potential links to malaria drug targets, offering a promising avenue for further research. This work combines machine learning and network-based techniques to enhance the prediction of essential metabolic genes in the *P*. *falciparum* genome-scale metabolic network, with implications for novel antimalarial strategies [2].

## Methods

### Datasets

The study used most recent GSMN model of *P*. *falciparum* (iAM_pf480) curated by Abdel-Haleem *et al*. [2], which is publicly available on BiGG (http://bigg.ucsd.edu/), a knowledge base GSMN model. iAM_pf480 contains 480 genes, 617 distinct metabolites, and 1083 reactions. Gene-protein-reaction (GPR) interactions involving 480 genes and 68% of all enzymatic processes included in the model [2]. The details on the iAM_Pf480 model are listed in Table 1.

**Table 1:** Description of iAM_Pf480 content [2].

**Metabolites**		**905**
	Unique Metabolites	617
	Cytoplasm	531
	Apicoplast	109
	Golgi	45
	Mitochondria	82
	Endoplasmic Reticulum	26
	Lysosome	9
	Extracellular	107
**Reactions**		**1082**
	Gene-Associated Reactions (Metabolic & Transport)	738 (68%)
	Exchange Reactions	92 (9%)
	Non-Gene Associated React (Metabolic)	76 (7%)
	Non-Gene Associated React (Transport)	160 (15%)
	Demand and Sink Reaction	16 (1%)
**Genes**		**409**

The iAM-Pf480 model encompasses six distinct subcellular locations, including the cytosol, mitochondria, Golgi apparatus, endoplasmic reticulum, food vacuole, and apicoplast. It compiles enzymes across all developmental stages of the model organism. Currently, iAM-Pf480 exhibits superior functionality compared to previous *P*. *falciparum* GSMN models, boasting a broader range of genomic content and a more extensive dataset of biochemical information, rendering it well-suited for in-depth investigations.

### Graph construction

The Mass Flow graph (MFGs) algorithm, developed by Beguerisse-Díaz et al. in 2018 [30], was utilized for constructing metabolic graphs by Freischem et al., [11]. This algorithm introduced a novel machine learning method to predict gene essentiality directly from wild-type flux distributions, without assuming the optimality of deletion strains. However, this approach has not been explored in pathogenic eukaryotic organisms, and the impact of other connectivity features on gene essentiality prediction has not been investigated [11]. Therefore, we adopt their implementation and expand the scope to include additional connectivity features not previously considered in their work. MFGs integrate flux balance analysis solutions with the stoichiometric matrix of GSMMs to construct a flux-weighted reaction-centric (F-WRC) metabolic graph.

FBA is a widely accepted approach to studying cell metabolism and essentiality studies. FBA computes the best steady-state flux distribution of a cell; the flux distribution specifies the cell phenotype [11,18,20,23]. It accepts a genome scale metabolic model as inputs, containing Stoichiometric matrix as ***S*** (i.e, a matrix containing metabolites as rows and reactions either being produced or consumed as columns). The objective of FBA is to find the solution flux vector v, that satisfies the mass balance equation given as *Z*.

Mathematically;

$$Max\;Z=C^{T}V$$ (1)

*Subject to*

$$\frac{dy}{dt}=SV=0$$ (2A)

$$v_{l}\leq v\leq v_{u}$$ (2B)

where *C* encodes the cell objective function, $\frac{dy}{dt}$ is the concentration of metabolite *y* with respect to time *t*,*v*_*l*_ and *v*_*u*_ are vectors containing the lower and upper limits on the fluxes of the reactions involved, respectively. Researchers can determine flux-flow of the cells under different environmental and genetic conditions by altering the reaction flux bounds [31,32]. FBA has been applied primarily in the studies of gene essentiality prediction via performing single/double gene and/reaction essentiality in silico simulations.

Further details on the theory and python script implementation of the expanded MFGs can be found in the (S1 File) attached to this study. We wrote a Python script to implement the extended Mass Flow graph (MFG) algorithm, generating an F-WRC graph from the iAM-Pf480 GSM model. This assumed aerobic growth with glucose as the sole carbon source, consistent with the experimental conditions used to construct the GSMM [2]. The resulting MFGs can be exported in both numpy and CSV file formats for subsequent analysis.

Below is the summarized algorithm that implements MFG.

**Step 1**: Input Data: iAM_pf480

Obtain stoichiometric matrix S and FBA flux vector v from the Genome-scale metabolic model.

**Step 2**: Calculate Reversibility Vector

Initialize an m-dimensional reversibility vector r, where m is the number of reactions. This vector is normally attached to the GSMM Model.

For each reaction j:

If reaction j is reversible:

$$r_{j}=1$$

Else:

$$r_{j}=0$$

**Step 3**: Construct *S*_2*m*_

Create an extended stoichiometric matrix *S*_2*m*_ by combining S and its negative counterpart.

Extend *S*_2*m*_ by adding identity matrices:

**Step 4**: Calculate $S_{2m}^{+}$ and $S_{2m}^{-}$

$$S_{2m}=[S\;-S]\left[\begin{array}{cc} l_{m} & 0\\ 0 & diag(r) \end{array}\right]$$ (3)

Calculate production, $S_{2m}^{+}$ and consumption, $S_{2m}^{-}$ matrices:

$$Consumption:\;S_{2m}^{+}=\frac{1}{2}(abs(S_{2m})+S_{2m})$$ (4)

$$Production:\;S_{2m}^{+}=\frac{1}{2}(abs(S_{2m})+S_{2m})$$ (5)

**Step 5**: Split Flux Vector

Split the flux vector v into two vectors, v*^+^ and v*^−^s:

$$V_{2m}^{*}=[v^{*+}\;v^{*-}]=\left[\begin{array}{c} abs(v)+v^{*}\\ abs(s)-v^{*} \end{array}\right]$$ (6)

**Step 6**: Calculate Production and Consumption Fluxes

Calculate production and consumption fluxes using S_2m_plus, S_2m_minus, v_plus, and v_minus:

$$j_{i}(v)=S_{2m}^{+}v_{2m}^{*}=S_{2m}^{-}v_{2m}^{*}$$ (7)

**Step 7**: Calculate the MFG Adjacency Matrix

Compute the MFG adjacency matrix M(v*) using the formula:

$$M(v^{*})={\left(S_{2m}^{+}V^{*}\right)}^{T}J_{v}^{\tau }(S_{2m}^{-}V^{*})$$ (8)

Where:


$$V^{*}=diag(v_{2m}^{*})$$

$$J_{v}=diag(j(v^{*}))\;and$$

$$\tau =the\;matrix\;pseudoinverse\;of\;J_{v}$$

**Step 8**: Return MFG Adjacency Matrix

The MFG adjacency matrix is the output of the algorithm.

Our implementation produced a weighted metabolic graph focused on reactions, comprising 505 reactions as nodes and 6217 edges derived from the iAM-Pf480 model.

To assign essential or non-essential labels to reaction nodes, data on the essentiality of *Plasmodium falciparum* genes from the **O**nline **GE**ne **E**ssentiality (OGEE) database was used. This database contains genes confirmed as essential or non-essential through various experimental techniques [33,34]. As the OGEE database provides gene-level essentiality data, there was a need to translate these labels into the reaction context. This required the application of gene-protein-reaction (GPR) Boolean rules included in the iAM-Pf480 genome-scale model, which describes the link between metabolic reactions to genes. It’s important to note that some reactions may not be associated with any gene, lacking an essentiality label. Consequently, such reactions were excluded after extracting graph features, leaving us with 330 reactions for analysis [11].

### Feature extraction

Metabolic Flow Graphs (MFGs) lack specific attributes for each reaction node in the graph. To address this, the graph was further exported for feature extraction. Node features were obtained using the COBRApy toolbox v0.26.3 with the glpk solver and the default iAM-Pf480 model objective function. Our study encompassed four categories of graph features: Node role analysis features (ReFeX and RolX), network centrality-based features, and adjacency-based features.

### Recursive feature extraction algorithm (ReFeX)

ReFeX, developed by Henderson and colleagues in 2011, is a valuable node role analysis tool for directed graph networks. It excels at extracting meaningful, transferable features from graph nodes, making it instrumental in identifying and classifying nodes based on their characteristics within a network. The algorithm’s key attributes—local, egonet, and recursive—offer various insights into nodes and their relationships. Local features, such as degree and total degree, reflect a node’s connectivity and centrality within the network. Egonet features focus on subgraphs formed by a node and its neighbours, providing information about the node’s influence within its immediate neighbourhood, including identifying hubs or bridges between different parts of the network [35,36].

### Role eXtraction (RolX)

In 2012, Henderson and colleagues introduced Role eXtraction (RolX), an unsupervised method for automatically deriving structural roles from directed networks [35]. RolX employs a mixed-membership strategy, distributing each node’s role across the detected roles. The process involves three key steps: recursive feature extraction (ReFeX), feature grouping, and model selection, with inspiration from Henderson et al. [35]. Utilizing a mixed-membership approach, RolX mechanically identifies roles within a graph. It employs nonnegative matrix factorization to approximate the node feature matrix V:

$$V_{n\times f}\approx G_{n\times r}\times F_{r\times f}$$ (9)

where entries *G*_*ij*_ quantify the membership of node *n*_*i*_ in role *r*_*j*_ and entries *F*_*jk*_ specify how a membership in role *r*_*j*_ contributes to the value of feature *F*_*jk*_. Given the number of roles denoted by r, RolX was applied. The rank r in this approximation is equal to the total number of roles. These two matrices efficiently compress V, if node roles summarize node activity in the network.

To extract these features, *GraphRole* (a PyPl package that implements ReFeX and RolX algorithms on directed graphs developed by Kaslovsky, 2019 [37]) was deployed on the graph to extract ReFeX and RolX. It specifically uses a mixed-membership assignment strategy to group the nodes into five separate roles (*r*1,*r*2,*r*3,…,*r*5) to form RolX feature matrix. These roles are denoted by the letters r1, r2, r3, …, and r5, respectively. The percentage representing the node’s contribution to each role was ascribed to each individual node.

### Network centrality-based features

Centrality-based features have gained recognition as a valid approach to characterizing essential metabolic genes. Several studies have explored the interplay between network topology and biological processes [5,38]. The centrality-lethality hypothesis in biological networks posits that central nodes are more likely to be vital for the overall well-being of the system [39], and numerous studies have investigated the connection between centrality and essentiality within biological networks [40]. Therefore, nodes with higher centralities are more likely to be indispensable for the network. From the flux-weighted network of iAM_Pf480, six topological metrics were extracted: PageRank, PageRank Percentage, Betweenness centrality, closeness centrality, Clustering Coefficient, and degree. This feature matrix, with 330 rows and 6 columns, was utilized as input for the machine-learning model to train and predict gene essentiality.

### Adjacency features

The adjacency matrix derived from the Metabolic Flux Graph (MFG) can be used as a node feature matrix for training machine learning models to predict gene essentiality, as discussed by Freischem et al. [11]. When creating a feature matrix from the adjacency matrix (M) of the MFG, it’s important to consider that solutions obtained through Flux Balance Analysis (FBA) often exhibit sparsity, with many reactions having zero flux, indicating non-essential genes as disconnected nodes in the MFG. Nodes disconnected at position "i" result in both the "i"th row and column of the adjacency matrix (*M*_*m*_) containing only zeros. By identifying the nonzero nodes as "k" in the adjacency matrix (*M*_*m*_) representing metabolites, a reduced matrix (*M*_*k*_) is formed by removing nodes associated with zero flux. This process results in the construction of the feature matrix *X* (*m*−*k in size*:

$$X_{m}=[M_{k}\;M_{k}^{T}]$$ (10)

In brief, four different feature sets were derived from the graph: ReFeX features, RolX features, adjacency matrix features, and topological/centrality features. Following feature extraction, some reactions—devoid of gene linkage and essentiality labels—were eliminated, leaving 330 reactions. The analysis involved data augmentation through a combination of ReFeX, RolX, and Centrality features to assess their impact on essentiality prediction. More specific details are available in Table 2.

**Table 2:** Various feature sets used in the experiment.

Dataset	No of Features
**RolX**	5
**ReFeX**	31
**Topological Features**	6
**Adjacency Features**	1010
**ReFeX&RolX**	36
**Topology&ReFeX**	37
**Topology&RolX**	11
**Topology&ReFeX&RolX**	42

### ML classifiers

**Fig 2:**
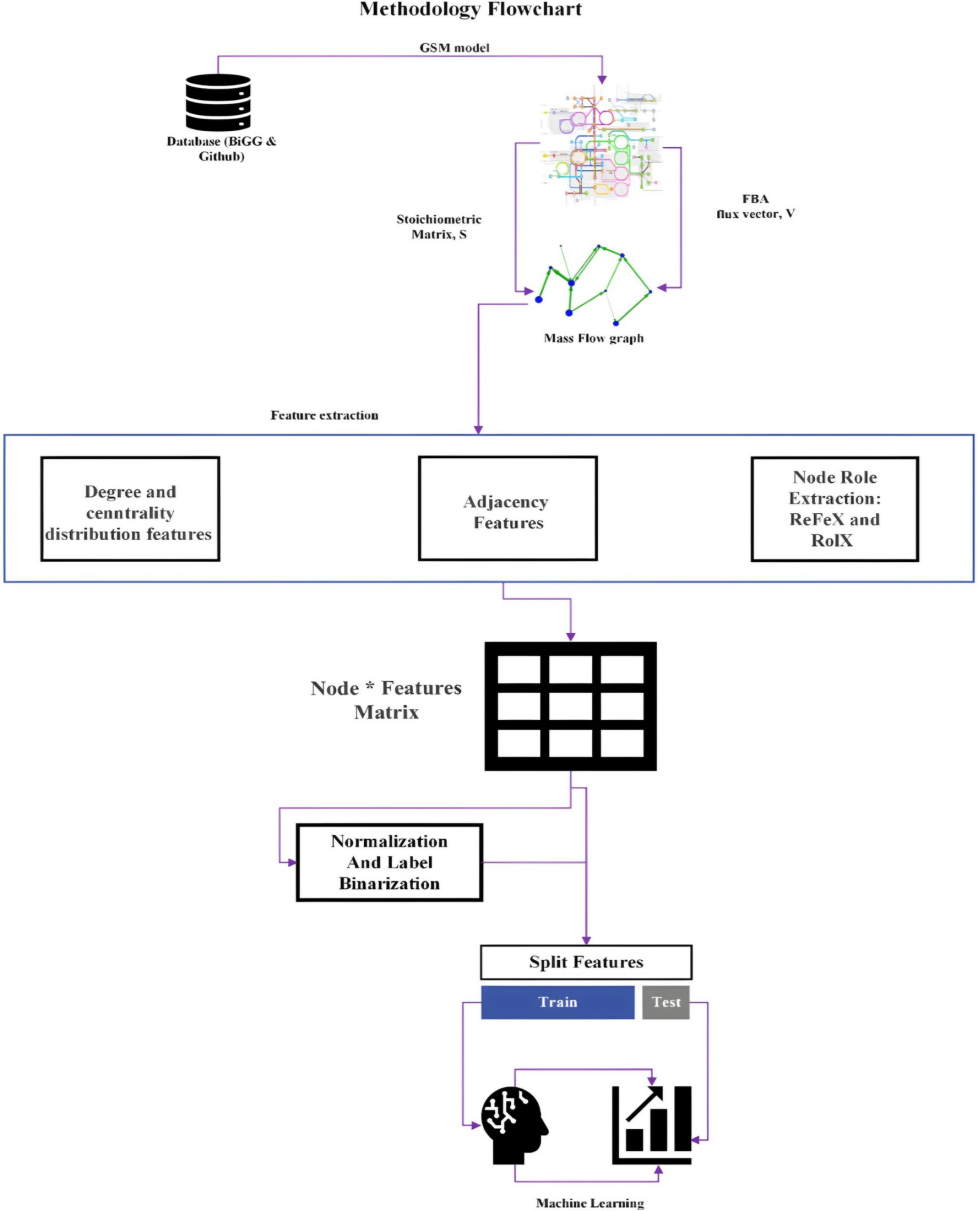
Machine learning pipeline. The models were trained on 80% of data, with the remaining 20% reserved as test set.

In this research a machine learning pipeline was designed, as depicted in Fig 2, to build binary classifiers for predicting essentiality labels. This pipeline utilised characteristics gathered from the mass flow graphs, employing the Python programming language and the Scikit-learn library. Several ML algorithms, including Support Vector Machine (SVM), Logistic Regression (LG), Random Forest (RF), Decision Tree (DT), k-nearest neighbour (kNN), and Naive Bayes (NB), were employed and evaluated for their performance across different datasets [5,10]. The study identified the most effective machine learning algorithm and the feature sets that yielded the highest prediction accuracy.

### Performance evaluation

This study examined the performance of the ML binary classifiers using the evaluation metrics discussed below.

#### 1. Accuracy

The accuracy is a performance matrix calculated using a confusion matrix. This accounts for the percentage of correctly predicted outcomes. $Accuracy=\frac{TP+TN}{TP+TN+FP+FN}.$ where TP = True Positive, TN = True Negative, FP = False Positive, FN = False Negative.

Accuracy is most suitable for cases of perfectly balanced data that must prove misleading in situations where our data are imbalanced.

#### 2. Precision

Precision is a measure of the number of predicted positive outcomes that are positive. It shows the number of correct positive predictions compared with the total number of positive predictions, $Precision=\frac{TP}{TP+FP}.$

#### 3. Recall/Specificity

Recall shows how many of the actual positive values are predicted to be positive. It shows how many correct positive predictions there are compared to how many positive cases there are in the entire dataset, $Recall=\frac{TP}{TP+FN}$.

#### 4. F1 Score

It is a balance between recall and accuracy. Its interval is [0,1]. This statistic often informs us of the classifier’s precision (number of cases properly classified) and robustness (absence of significant number of missed instances), $F1\;Score=\frac{2}{\frac{1}{Precision}+\frac{1}{Recall}}.$

#### 5. Area Under the curve of the Receiver Operating Characteristic (AUROC)

The AUROC (Area Under the Receiver Operating Characteristic Curve) measures a model’s performance by quantifying the area under a graph that charts the False Positive Rate (FPR) against the True Positive Rate (TPR) at different classification thresholds for a given problem. A higher AUROC score, approaching 1.0, reflects superior performance, with a lower FPR and a higher TPR. In essence, a higher AUROC score indicates better model performance, making it a valuable metric for evaluating classification models.

### Computational power

In this study, all experiments were conducted in Python 3, utilising various libraries, including *scikit-learn* [41], *networkX* [42], *MFG_updatepy* (a modified script of MFGpy for automated MFG graph generation and centrality features) [11], *GraphRole* (utilized for automated Recursive Feature Extraction and node role analysis) [37], and *COBRApy* [43]. The algorithms and scripts ran on a personal computer equipped with an AMD Core CPU operating at 2.70GHz and 16 GB of RAM.

## Results and discussions

### Classification algorithms on different graph-based feature sets

The model evaluation began with training six binary classification models, optimising their hyperparameters through 5-fold cross-validation. Cross-validation involves splitting the data into five subsets (folds) and training the model on four while evaluating it on the remaining fold, repeating this process five times for a reliable performance estimate. The model’s performance was examined using five common metrics: Area under the Receiver Operating Characteristic curve (AuROC), Accuracy, Precision, Recall, and F1-score, providing insights into classification accuracy.

**Fig 3:**
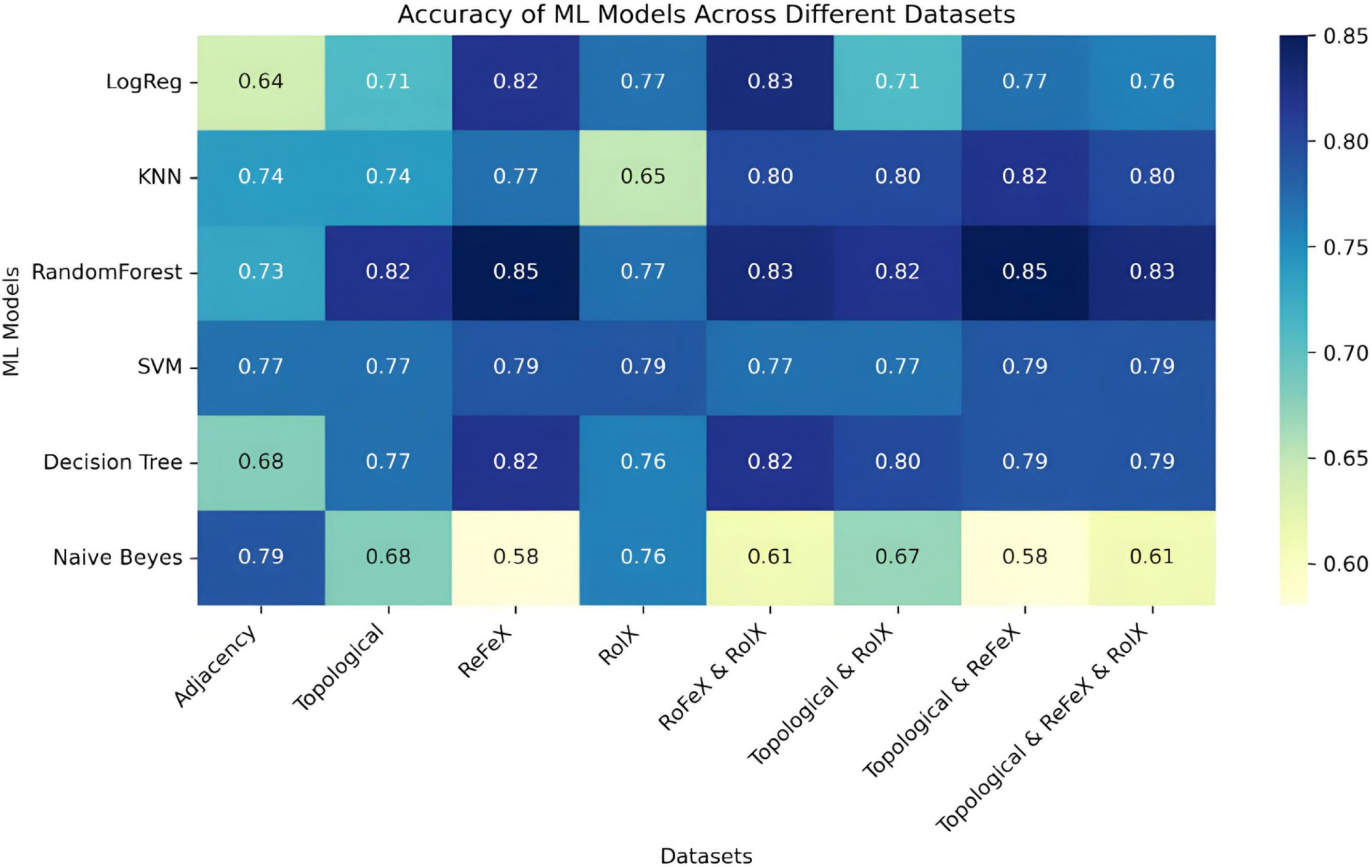
Accuracy heatmap of six machine learning models across different datasets. The Machine learning models consist of Naive Bayes, Decision Trees, Support Vector Machine, Random Forest, k-Nearest Neighbour, and Logistic Regression showcasing their accuracy metrics.

The model evaluation results, along with optimised hyperparameters, are summarised in supplementary Table 1 in S1 File. A heatmap in Fig 3 as shown below depicts the performance ML models across different datasets showing their accuracy metrics. The datasets consist of 330 reactions, with 258 (78%) deemed essential and 72 (22%) classified as non-essential. Considering the dataset’s class imbalance, the weighted average for precision, recall, and F1-score was used to account for uneven class distribution. This approach ensures a balanced evaluation. An 80% portion was allocated as the training set, while the remaining 20% was designated as the test set. The recorded results reflect the performance of models trained on 80% of the available reactions, with the remaining 20% reserved as a held-out/test set to assess the models’ ability to generalize to unseen data without bias.

**Fig 4:**
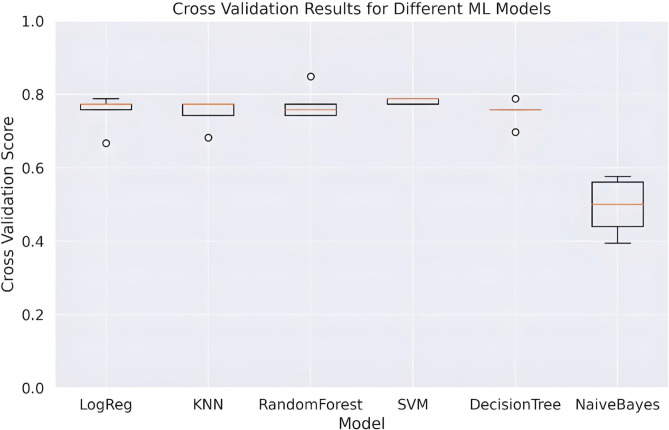
Box plot depicting the 5-fold cross-validation results of various machine learning models on the ReFeX dataset. The plot highlights the performance spread and stability across different models, with RandomForest and SVM showing more consistent results compared to Naive Bayes, Decision Tree, and KNN.

To assess the performance of different machine learning models and examine potential overfitting, we generated a box plot illustrating the 5-fold cross-validation results using accuracy metrics on the ReFeX dataset, as depicted in Fig 4. Our analysis revealed that Random Forest and SVM consistently exhibited narrower performance spreads across folds, suggesting more stable performance. Conversely, Naive Bayes displayed a wide spread of scores, indicating potential overfitting or instability. Decision Tree and KNN also showed variability in scores, highlighting differing performance across subsets of data.

**Fig 5:**
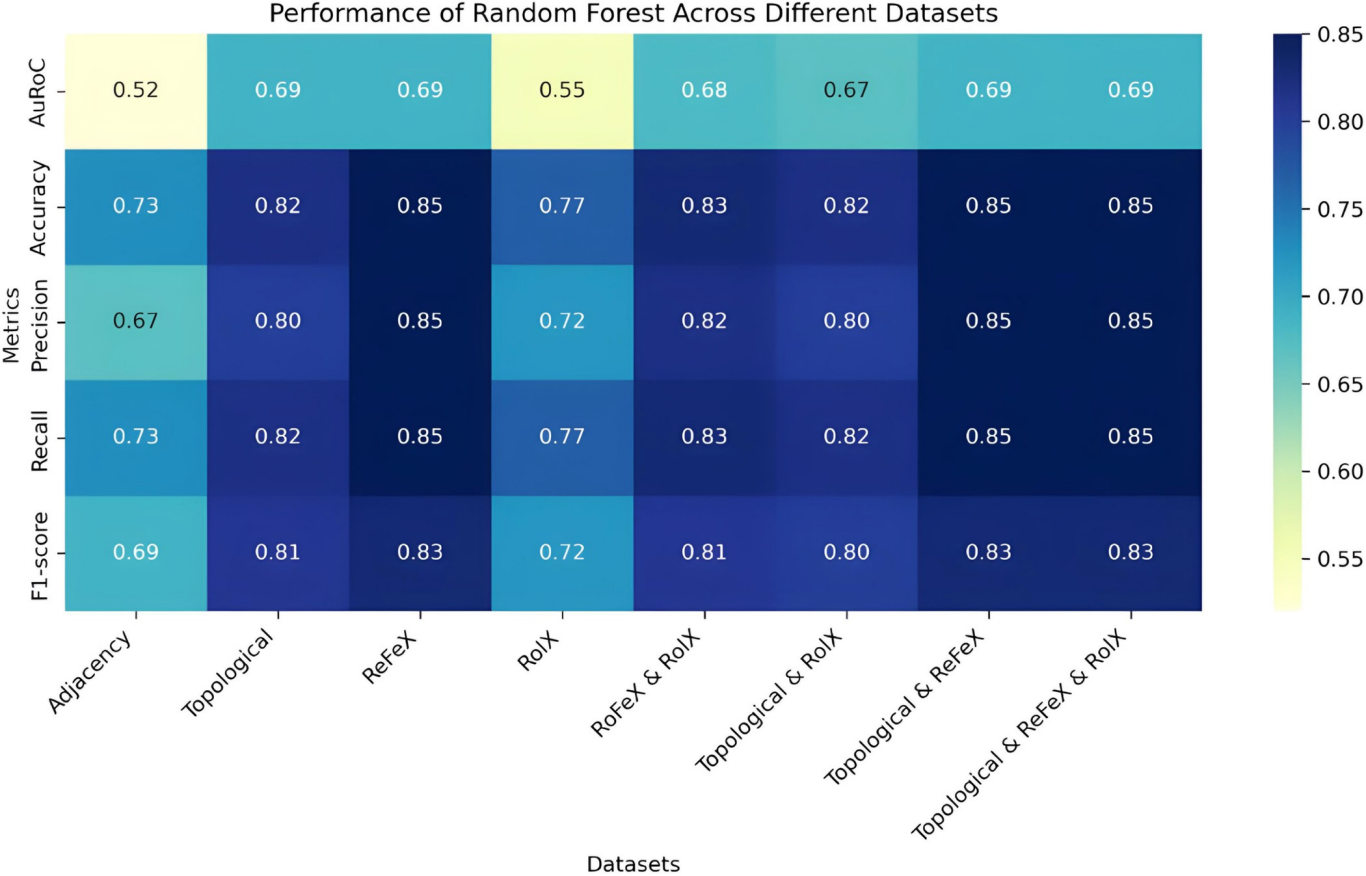
Performance of random forest across all datasets. This shows the performance of RF across all the datasets, with above 0.83 for all the metrics and 0.69 AuROC in ReFeX, Combined Topological&ReFeX, and Topological&ReFeX&RolX feature sets.

In the overall assessment, the Random Forest classifier with 500 trees and a maximum depth of 42 consistently yielded the best results across various feature sets, especially excelling in the ReFeX feature set. Information gain guided the best tree splits, and most feature sets (except Adjacency and RolX) utilized log2 (2k) features. The model achieved an 85% accuracy on the ReFeX test dataset. Notably, the model demonstrated an 85% accuracy and an 83% recall rate when applied to the ReFeX, Combined Topological&ReFeX, and Topological&ReFeX&RolX feature sets. It’s worth highlighting that ReFeX significantly influenced Random Forest’s performance in feature combinations containing the ReFeX set. Fig 5 is a heatmap that shows the performance of RF across all the datasets used in these experiments.

**Fig 6:**
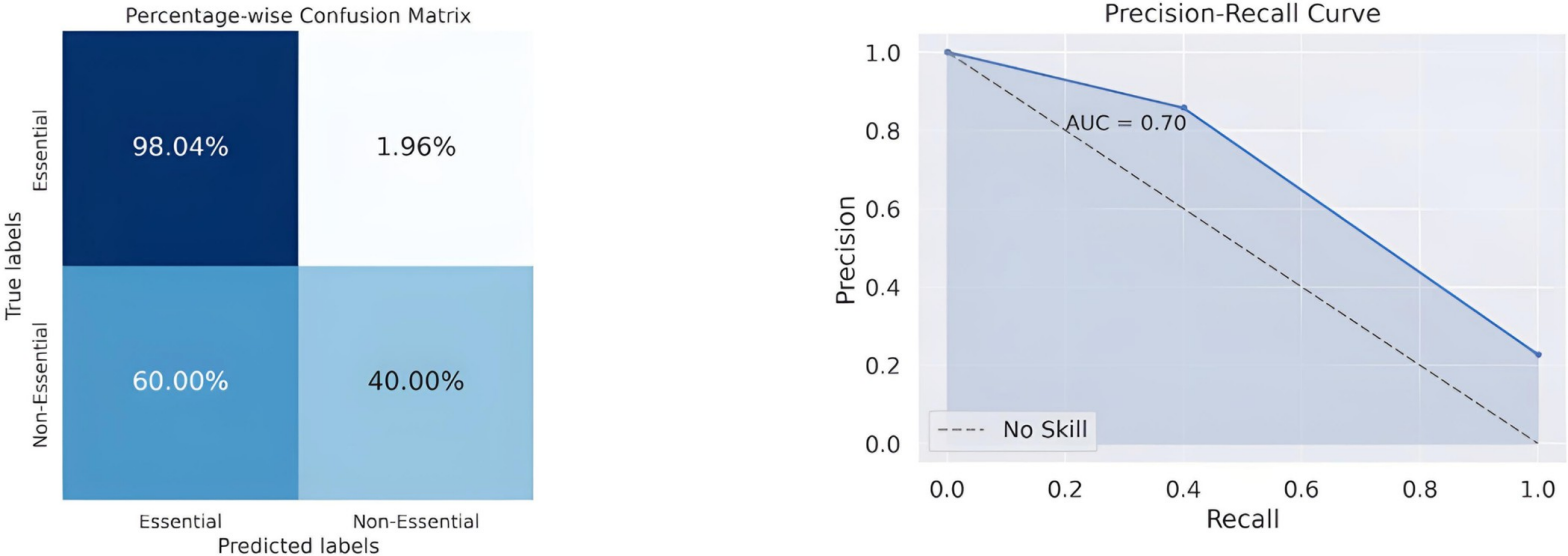
**Gene essentiality prediction in *Plasmodium falciparum* (iAM_Pf480)** (A) Normalised Percentage-wise Confusion Matrix of Random Forest on ReFeX Features and (B) Precision-Recall Curve of Random Forest. We see AUC = 0.7 indicates that the model’s ability to differentiate between the positive and negative classes is of moderate strength.

The normalized percentage-wise confusion matrix (Fig 6A) was examined on the test dataset, and it suggested that the classifier is relatively bad at predicting the non-essential reactions (with an accuracy of 40%), but it shows near state-of-the-art accuracy for essential genes (with an accuracy of 98.04%). This discrepancy could be explained by the fact that the non-essential reactions are not as well represented in the dataset as the essential reactions are. Comparing the RF model’s accuracy of 85% on the ReFeX dataset with the baseline "no skill" accuracy of 77% (Fig 6B) inferred from the confusion matrix (Fig 6A) indicates that the ML model performs significantly better than the naive classifier.

### Comparative studies with FBA analysis

This study employed the COBRApy library to conduct Single Reaction Deletion analysis on the Genome-Scale Metabolic (GSM) model of *Plasmodium falciparum*. The analysis focused on the performance of FBA on reactions represented in the flux-weighted reaction-centric graph we constructed, with the primary objective of evaluating the accuracy of Flux Balance Analysis (FBA) in predicting reaction nodes. The findings are reported in a confusion matrix, providing a detailed breakdown of FBA’s performance compared to actual reaction labels. The confusion matrix included True Positive (TP), True Negative (TN), False Positive (FP), and False Negative (FN) categories (Table 3).

**Table 3:** Confusion matrix on FBA predictions on the dataset.

		Traditional FBA Predicted		RF-ML Predicted	
**True Labels**		Essential	Non-Essential	Essential	Non-Essential
	Essential	138	120	245	13
	Non-Essential	17	55	13	10
	**Accuracy**		**0.59**		**0.77**

**Fig 7:**
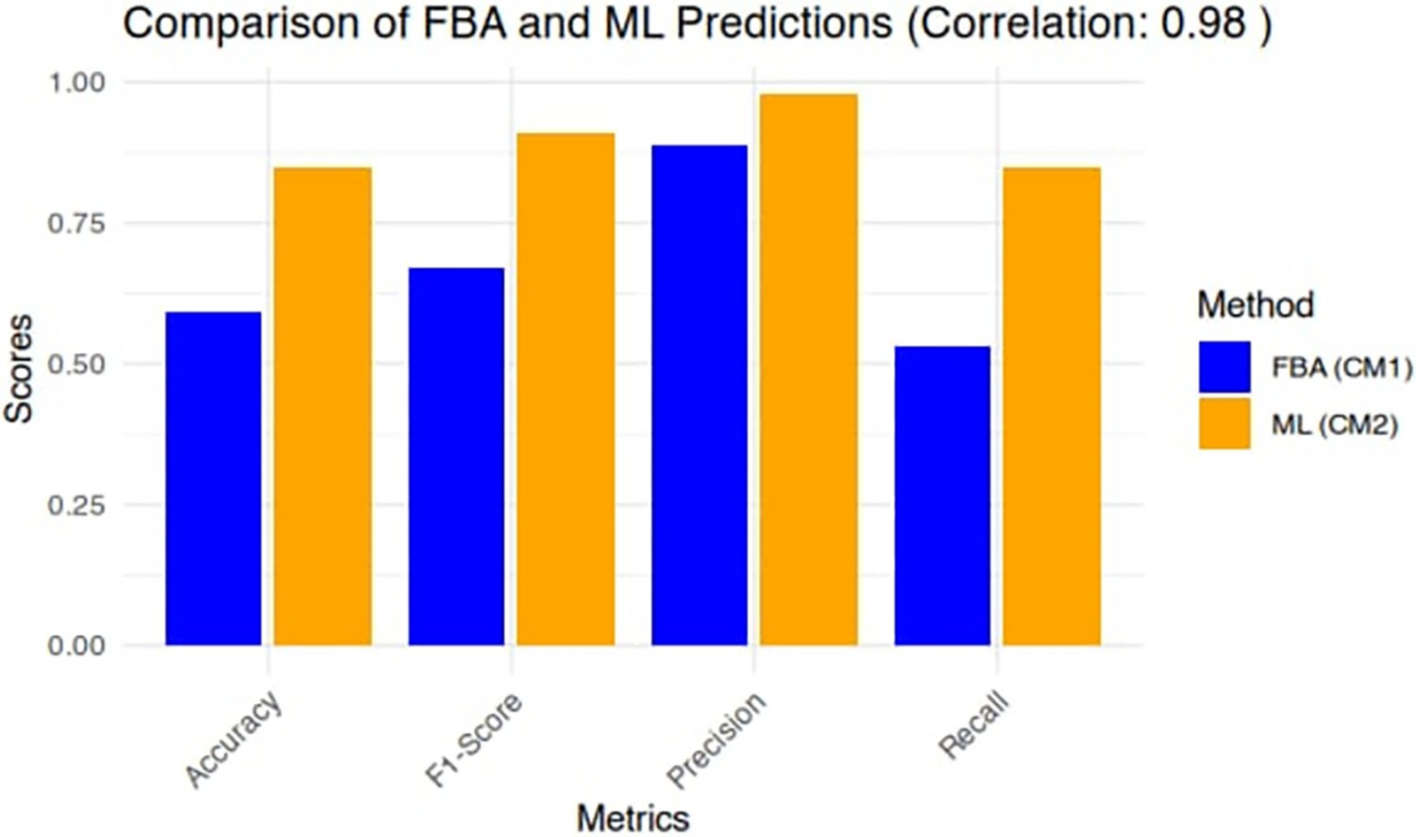
Bar chart comparing the performance of the traditional FBA method and the best-performing Random Forest (RF) ML method. The RF model outperforms Traditional FBA across all evaluated metrics on the dataset.

According to the accuracy of FBA predictions, 138 out of 258 essential reactions were correctly identified by FBA (TP), but it mislabelled 55 non-essential reactions as essential (FP) and 120 essential reactions as non-essential (FN) resulting in an accuracy of 0.59. In contrast, the best performing machine learning (ML) model used in this study achieved better performance with an accuracy of 0.77 on all reaction sets that were included in our study, surpassing the FBA model in terms of accuracy on the same reaction set. Fig 7 compares the confusion matrix of the traditional FBA and basic matrices; F-Score, Accuracy, Precision and Recall.

These results indicate that while FBA offers insights into reaction essentiality, it may not be as precise as the ML model used here. The improved accuracy of the ML model suggests its ability to classify and predict essential reactions and their respective essential genes, enhancing our understanding of the organism’s metabolic behaviour more accurately. Overall, these findings suggest that integrating both FBA and ML techniques could provide a more comprehensive and accurate analysis of metabolic essentiality, assisting researchers in gaining deeper insights into the organism’s metabolic network.

### Biological findings

In comparing the machine learning model’s predictions to the OGEE node label information, the study revealed 9 genes marked as nonessential but predicted as essential (False Positives). To gain deeper insights, a literature survey was conducted to explore the essentiality of these genes and found that four of these have been considered potential drug targets. The specifics of these genes are listed in Table 4. Further discussions regarding the experimental evidence gathered from the literature and the potential applications of these genes in malaria drug discovery can be found in the supplementary report in S1 File.

**Table 4:** List of false positive prediction (genes labelled as non-essential but predicted as essential).

Reaction/Enzyme	Gene	Binary labels	ML Prediction	Stage in Pf Cycle
ACONTb (aconitate hydratase (IRP))	PF3D7_1342100	NE	E	Plays a significant role in TCA, at the asexual and gametocyte stage of the parasite[44].
MAN6PI	PF3D7_0801800	NE	E	Inhibits the growth of *Plasmodium* parasites during the blood stage [45].
PPPGO6m	PF3D7_1028100	NE	E	Disrupt energy production at the liver stage [46]
PYNP2r	PF3D7_0513300	NE	E	Salvages purines for its rapid replication within red blood cells in the Blood stage [47].
TMPPP	PF3D7_0614000	NE	E	No Evidence
THBPT4ACAMDASE	PF3D7_1108300	NE	E	No Evidence
CITtcm	PF3D7_1223800	NE	E	No Evidence
DHORTS	PF3D7_1472900	NE	E	No Evidence
SUCOAS1m	(PF3D7_1437700 or PF3D7_1431600) and PF3D7_1108500	NE	E	No Evidence

We present a discussion of experimental evidence found in the literature regarding specific genes and their potential applications in malaria drug discovery:

1. Gene PF3D7_1342100 encodes for Aconitase hydratase (IRP), an enzyme responsible for catalysing the stereo-specific isomerization of citrate to isocitrate via cis-aconitate in the tricarboxylic acid cycle. A study conducted by Ke *et al*. [44] revealed that this gene plays a crucial role in the Tricarboxylic Acid Cycle in the mitochondrion of *Plasmodium falciparum*. Knocking out this gene resulted in the parasite’s inability to fully utilise glucose nutrients in the TCA cycle, affecting its carbon source. As a consequence, the parasite could not mature into gametocytes, hindering gamete formation. This study provides valuable experimental evidence to investigate further [44].
2. Gene PF3D7_0801800 codes for mannose-6-phosphate isomerase, which was investigated in *Plasmodium berghei*, a pathogen responsible for cerebral malaria in rodents. Lv *et al*. [45] found that administering D-mannose to *Plasmodium berghei*-infected mice resulted in weight loss and reduced parasitemia without noticeable side effects. Their findings suggest that mannose prevents *Plasmodium* infection by regulating multiple host immune responses and could serve as a potential strategy for facilitating malaria treatment [45].
3. Gene PF3D7_1028100 encodes for protoporphyrinogen oxidase (PfPPO), localised in the mitochondria and active under anaerobic conditions. PfPPO depends on electron transport chain (ETC) acceptors for its activity. Notably, ETC inhibitors, such as atovaquone and antimycin, inhibit the enzyme’s function. Atovaquone, a known parasite dihydroorotate dehydrogenase inhibitor, inhibits heme synthesis in *P*. *falciparum* culture and has been used to design Atovaquone-proguanil, an antimalarial drug [46,48].
4. Gene PF3D7_0513300 encodes for purine nucleoside phosphorylase (PfPNP), representing a potential target for antimalarial drug design. Inhibition of PfPNP has been shown to effectively kill malaria parasites both in vitro and in vivo [47]. However, currently known inhibitors, immucillins, are orally available and exhibit low toxicity to animals and humans. Yet, none of these compounds have entered clinical trials for malaria treatment [49,50].
5. For the remaining genes (PF3D7_0614000, PF3D7_1108300, PF3D7_1223800, PF3D7_1472900, PF3D7_1437700 (or PF3D7_1431600), and PF3D7_1108500), there is no literature evidence suggesting their direct biological relevance in malaria drug discovery. Further research is required to gain insight into their potential roles in the malaria parasite’s metabolism and pathogenesis.

## Conclusion

In conclusion, the prediction of metabolically essential genes remains a difficult challenge, particularly in the context of eukaryotic pathogenic organisms such as *Plasmodium falciparum*, which causes most malaria cases. While significant progress has been made in the study of prokaryotic species, there is still a great deal of work to be done. Various approaches, spanning from sequence features to network-based methods, have been utilised in prior research [12,51].

Numerous of these studies have focused on prokaryotic organisms and have represented metabolic networks as unweighted, undirected graphs, which do not adequately characterise the flow and flux-weighted nature of metabolic networks. The objective of this study was to extend the application of machine learning to predict essential genes from a reaction-centric metabolic network in the eukaryotic organism *Plasmodium falciparum*. This approach is intended to serve as a precedent for applying similar methodologies in other eukaryotic systems, marking the first instance of such work in this context, to the best of our knowledge. While extensive work has been done on prokaryotes, this study pioneers the application of this approach in eukaryotes. This graph was constructed using the Mass Flow graph algorithm proposed by Beguerisse-Díaz et al., [30], and we selected the most up-to-date Genome-Scale Metabolic Model (GSMM), iAM-Pf480 from the BiGG database, which includes reactions from six distinct subcellular locations (cytosol, mitochondria, Golgi apparatus, endoplasmic reticulum, food vacuole, and apicoplast) and enzymes across all developmental stages of the organism along with essentiality data from the OGEE database.

To predict metabolic essential genes, performance evaluation was carried out on several machine learning algorithms employing features derived from local, neighbourhood, and global network properties, including adjacency matrix features. Compared to experimentally validated datasets, six centrality features and ReFeX features derived from our graph demonstrated high predictability. Random Forest obtained the highest predictability among the tested machine learning algorithms and successfully identified essential genes.

In addition, a comparison of the performance of our best machine learning model to that of traditional FBA was carried out and discovered that our model performed better at classifying essential reaction nodes, using the node OGEE essentiality labels. This research enhances our understanding of metabolic networks and their role in determining the essentiality of genes. Notably, this approach identified genes categorised as non-essential in the OGEE database but predicted that they were essential. A literature survey conducted shows that numerous of these genes have potential as drug targets for the treatment of malaria, indicating intriguing avenues for future research.

However, it is important to acknowledge the limitations of this study, including its narrow focus on *Plasmodium falciparum*, which restricts its generalizability. Further investigation is needed to assess the applicability of this approach to other eukaryotic organisms and apicomplexan parasites, such as *Plasmodium berghei or T*. *gondii*. Additionally, this research utilized the most updated Genome-Scale Metabolic Model (GSMM) of *Plasmodium falciparum* constructed by Abdel-Haleem et al. in 2018 [2], which, to the best of our knowledge, remains the most current model. Future research should consider the quality of GSMMs, as it significantly influences predictions of metabolic essentiality. Recent studies, such as Hasibi et al. [52], have highlighted the potential of deep learning to enhance machine learning models for gene essentiality prediction. By integrating graph neural network approaches with Flux Balance Analysis (FBA), researchers can train knock-out fitness data without assuming the optimality of deletion strains, effectively leveraging the inherent graph structure of cellular metabolism.

## Supporting information

S1 File Mass Flow graph algorithm and experimental report. This contains a supplementary report on the Mass Flow graph algorithm and a table of the full report of experimental results.
(DOCX)

## References

[1] Carey MA, Papin JA, Guler JL. Novel Plasmodium falciparum metabolic network reconstruction identifies shifts associated with clinical antimalarial resistance. BMC Genomics. 2017;18. doi: 10.1186/s12864-017-3905-1 28724354 PMC5518114

[2] Abdel-Haleem AM, Hefzi H, Mineta K, Gao X, Gojobori T, Palsson BO, et al. Functional interrogation of Plasmodium genus metabolism identifies species- and stage-specific differences in nutrient essentiality and drug targeting. PLOS Comput Biol. 2018;14: e1005895. doi: 10.1371/journal.pcbi.1005895 29300748 PMC5771636

[3] World Health Organization. Geneva: World Health Organization; 2023. Licence: CC BY-NC-SA 3.0 IGO.; 2023.

[4] Xu T, Wang S, Ma T, Dong Y, Ashby CR, Hao G-F. The identification of essential cellular genes is critical for validating drug targets. Drug Discov Today. 2024;29: 104215. doi: 10.1016/j.drudis.2024.104215 39428084

[5] Li X, Li W, Zeng M, Zheng R, Li M. Network-based methods for predicting essential genes or proteins: A survey. Brief Bioinform. 2019;21: 566–583. doi: 10.1093/bib/bbz017 30776072

[6] Plata G, Hsiao T, Olszewski KL, Llinás M, Vitkup D. Reconstruction and flux‐balance analysis of the Plasmodium falciparum metabolic network. Mol Syst Biol. 2010;6: 408. doi: 10.1038/msb.2010.60 20823846 PMC2964117

[7] Pacheco MP, Bintener T, Ternes D, Kulms D, Haan S, Letellier E, et al. Identifying and targeting cancer-specific metabolism with network-based drug target prediction. EBioMedicine. 2019;43: 98–106. doi: 10.1016/j.ebiom.2019.04.046 31126892 PMC6558238

[8] Nurlaila I, Irawati W, Purwandari K, Pardamean B. K-Means Clustering Model to Discriminate Copper-Resistant Bacteria as Bioremediation Agents. Procedia Comput Sci. 179: 804–812. Available: https://www.sciencedirect.com/science/article/pii/S1877050921000880.

[9] Aromolaran O, Beder T, Oswald M, Oyelade J, Adebiyi E, Koenig R. Essential gene prediction in Drosophila melanogaster using machine learning approaches based on sequence and functional features. Comput Struct Biotechnol J. 18: 612–621. Available: https://www.sciencedirect.com/science/article/pii/S2001037019305628. doi: 10.1016/j.csbj.2020.02.022 32257045 PMC7096750

[10] Nandi S, Subramanian A, Sarkar RR. An integrative machine learning strategy for improved prediction of essential genes in Escherichia coli metabolism using flux-coupled features. Mol Biosyst. 2017;13: 1584–1596. doi: 10.1039/c7mb00234c 28671706

[11] Freischem LJ, Barahona M, Oyarzún DA. Prediction of gene essentiality using machine learning and genome-scale metabolic models. Cold Spring Harbor Laboratory; 2022 Mar. doi: 10.1101/2022.03.31.486520

[12] Nandi S, Ganguli P, Sarkar RR. Essential gene prediction using limited gene essentiality information–An integrative semi-supervised machine learning strategy. PLOS ONE. 2020;15: e0242943. doi: 10.1371/journal.pone.0242943 33253254 PMC7703937

[13] Ferreira AEN, Sousa Silva M, Cordeiro C. Metabolic network inference from time series. Systems Medicine. Elsevier; 2021. pp. 127–133. doi: 10.1016/b978-0-12-801238-3.11347–9

[14] Shen Y, Liu J, Estiu G, Isin B, Ahn Y-Y, Lee D-S, et al. Blueprint for antimicrobial hit discovery targeting metabolic networks. Proc Natl Acad Sci. 2010;107: 1082–1087. doi: 10.1073/pnas.0909181107 20080587 PMC2824290

[15] Chiappino-Pepe A, Pandey V, Billker O. Genome reconstructions of metabolism of Plasmodium RBC and liver stages. Curr Opin Microbiol. 2021;63: 259–266. doi: 10.1016/j.mib.2021.08.006 34461385

[16] Iranzadeh A, Mulder NJ. Bacterial pan-genomics. Microbial Genomics in Sustainable Agroecosystems. Singapore: Springer Singapore; 2019. pp. 21–38. doi: 10.1007/978-981-13-8739-5_2

[17] Hameri T, Fengos G, Ataman M, Miskovic L, Hatzimanikatis V. Kinetic models of metabolism that consider alternative steady-state solutions of intracellular fluxes and concentrations. Metab Eng. 2019;52: 29–41. doi: 10.1016/j.ymben.2018.10.005 30455161

[18] Wu SG, Wang Y, Jiang W, Oyetunde T, Yao R, Zhang X, et al. Rapid prediction of bacterial heterotrophic fluxomics using machine learning and constraint programming. PLOS Comput Biol. 2016;12: e1004838. doi: 10.1371/journal.pcbi.1004838 27092947 PMC4836714

[19] Bordbar A, Monk JM, King ZA, Palsson BO. Constraint-based models predict metabolic and associated cellular functions. Nat Rev Genet. 2014;15: 107–120. doi: 10.1038/nrg3643 24430943

[20] Gatto F, Miess H, Schulze A, Nielsen J. Flux balance analysis predicts essential genes in clear cell renal cell carcinoma metabolism. Sci Rep. 2015;5. doi: 10.1038/srep10738 26040780 PMC4603759

[21] Dusad V, Thiel D, Barahona M, Keun HC, Oyarzún DA. Opportunities at the interface of network science and metabolic modeling. Front Bioeng Biotechnol. 2021;8. doi: 10.3389/fbioe.2020.591049 33569373 PMC7868444

[22] Machicao J, Craighero F, Maspero D, Angaroni F, Damiani C, Graudenzi A, et al. On the use of topological features of metabolic networks for the classification of cancer samples. Curr Genomics. 2021;22: 88–97. doi: 10.2174/1389202922666210301084151 34220296 PMC8188584

[23] Sahu A, Blätke M-A, Szymański JJ, Töpfer N. Advances in flux balance analysis by integrating machine learning and mechanism-based models. Comput Struct Biotechnol J. 2021;19: 4626–4640. doi: 10.1016/j.csbj.2021.08.004 34471504 PMC8382995

[24] Cheng J, Wu W, Zhang Y, Li X, Jiang X, Wei G, et al. A new computational strategy for predicting essential genes. BMC Genomics. 2013;14. doi: 10.1186/1471-2164-14-910 24359534 PMC3880044

[25] Aromolaran O, Aromolaran D, Isewon I, Oyelade J. Machine learning approach to gene essentiality prediction: A review. Brief Bioinform. 2021;22. doi: 10.1093/bib/bbab128 33842944

[26] Vijayakumar S, Rahman PKSM Angione C. A hybrid flux balance analysis and machine learning pipeline elucidates metabolic adaptation in cyanobacteria. iScience. 2020;23: 101818. doi: 10.1016/j.isci.2020.101818 33354660 PMC7744713

[27] Yu Y, Yang L, Liu Z, Zhu C. Gene essentiality prediction based on fractal features and machine learning. Mol Biosyst. 2017;13: 577–584. doi: 10.1039/c6mb00806b 28145541

[28] Oyelade J, Isewon I, Uwoghiren E, Aromolaran O, Oladipupo O. In Silico Knockout Screening of Plasmodium falciparum Reactions and Prediction of Novel Essential Reactions by Analysing the Metabolic Network. BioMed Res Int. 2018;2018: 1–11. doi: 10.1155/2018/8985718 29789805 PMC5896307

[29] Plaimas Eils, König. Identifying essential genes in bacterial metabolic networks with machine learning methods. BMC Syst Biol. 2010;4: 1–16. doi: 10.1186/1752-0509-4-56 20438628 PMC2874528

[30] Beguerisse-Díaz M, Bosque G, Oyarzún D, Picó J, Barahona M. Flux-dependent graphs for metabolic networks. Npj Syst Biol Appl. 2018;4. doi: 10.1038/s41540-018-0067-y 30131869 PMC6092364

[31] Martins Conde P do R, Sauter T Pfau T Constraint based modeling going multicellular. Front Mol Biosci. 2016;3. doi: 10.3389/fmolb.2016.00003 26904548 PMC4748834

[32] Yasemi M, Jolicoeur M. Modelling Cell Metabolism: A Review on Constraint-Based Steady-State and Kinetic Approaches. Processes. 2021;9: 322. doi: 10.3390/pr9020322

[33] Chen W-H, Minguez P, Lercher MJ, Bork P. OGEE: an online gene essentiality database. Nucleic Acids Res. 2012;40: D901–906. doi: 10.1093/nar/gkr986 22075992 PMC3245054

[34] Gurumayum S, Jiang P, Hao X, Campos TL, Young ND, Korhonen PK, et al. OGEE v3: Online GEne Essentiality database with increased coverage of organisms and human cell lines. Nucleic Acids Res. 2020;49: D998–D1003. doi: 10.1093/nar/gkaa884 33084874 PMC7779042

[35] Henderson K, Gallagher B, Eliassi-Rad T, Tong H, Basu S, Akoglu L, et al. RolX. Proceedings of the 18th ACM SIGKDD international conference on Knowledge discovery and data mining. New York, NY, USA: ACM; 2012. doi: 10.1145/2339530.2339723

[36] Henderson K, Gallagher B, Li L, Akoglu L, Eliassi-Rad T, Tong H, et al. It’s who you know. Proceedings of the 17th ACM SIGKDD international conference on Knowledge discovery and data mining. New York, NY, USA: ACM; 2011. doi: 10.1145/2020408.2020512

[37] dkaslovsky. GitHub—dkaslovsky/GraphRole: Automatic feature extraction and node role assignment for transfer learning on graphs (ReFeX & RolX). 2023. Available: https://github.com/dkaslovsky/GraphRole.

[38] Kwon DH, Hwang JS, Kim SG, Jang YE, Shin TH, Lee G. Cerebrospinal fluid metabolome in parkinson’s disease and multiple system atrophy. Int J Mol Sci. 2022;23: 1879. doi: 10.3390/ijms23031879 35163800 PMC8836409

[39] Azhagesan K, Ravindran B, Raman K. Network-based features enable prediction of essential genes across diverse organisms. PloS One. 2018;13: e0208722. doi: 10.1371/journal.pone.0208722 30543651 PMC6292609

[40] Kim K, Kang M, Cho S-H, Yoo E, Kim U-G, Cho S, et al. Minireview: Engineering evolution to reconfigure phenotypic traits in microbes for biotechnological applications. Comput Struct Biotechnol J. 21: 563–573. Available: https://www.sciencedirect.com/science/article/pii/S2001037022005992. doi: 10.1016/j.csbj.2022.12.042 36659921 PMC9816911

[41] Pedregosa F, Varoquaux G, Gramfort A, Michel V, Thirion B, Grisel O, et al. Scikit-learn: Machine Learning in Python. J Mach Learn Res. 2011;12: 2825–2830. Available: http://jmlr.org/papers/v12/pedregosa11a.html.

[42] Hagberg AA, Schult DA, Swart PJ. Exploring Network Structure, Dynamics, and Function using NetworkX. In: Varoquaux G, Vaught T, Millman J, editors. Proceedings of the 7th Python in Science Conference. Pasadena, CA USA; 2008. pp. 11–15.

[43] Ebrahim A, Lerman JA, Palsson BO, Hyduke DR. COBRApy: COnstraints-Based reconstruction and analysis for python. BMC Syst Biol. 2013;7. doi: 10.1186/1752-0509-7-74 23927696 PMC3751080

[44] Ke H, Lewis IA, Morrisey JM, McLean KJ, Ganesan SM, Painter HJ, et al. Genetic Investigation of Tricarboxylic Acid Metabolism during the Plasmodium falciparum Life Cycle. Cell Rep. 2015;11: 164–174. doi: 10.1016/j.celrep.2015.03.011 25843709 PMC4394047

[45] Lv L, Xu Z, Zhao M, Gao J, Jiang R, Wang Q, et al. Mannose inhibits Plasmodium parasite growth and cerebral malaria development via regulation of host immune responses. Front Immunol. 2022;13. 10.3389/fimmu.2022.859228 PMC9546034 36211381

[46] Nagaraj VA, Arumugam R, Prasad D, Rangarajan PN, Padmanaban G. Protoporphyrinogen IX oxidase from Plasmodium falciparum is anaerobic and is localized to the mitochondrion. Mol Biochem Parasitol. 2010;174: 44–52. doi: 10.1016/j.molbiopara.2010.06.012 20603160

[47] Dziekan JM, Yu H, Chen D, Dai L, Wirjanata G, Larsson A, et al. Identifying purine nucleoside phosphorylase as the target of quinine using cellular thermal shift assay. Sci Transl Med. 2019;11. 10.1126/scitranslmed.aau3174 30602534

[48] Nixon GL, Moss DM, Shone AE, Lalloo DG, Fisher N, O’Neill PM, et al. Antimalarial pharmacology and therapeutics of atovaquone. J Antimicrob Chemother. 2013;68: 977–985. doi: 10.1093/jac/dks504 23292347 PMC4344550

[49] Holanda RJ, Deves C, Moreira-Dill LS, Guimar∼ aes CL, Marttinelli LKB, Fernandes CFC, et al. Plasmodium falciparum purine nucleoside phosphorylase as a model in the search for new inhibitors by high throughput screening. Int J Biol Macromol. 2020;165: 1832–1841. doi: 10.1016/j.ijbiomac.2020.10.062 33075341

[50] Kagami LP, das Neves GM, Rodrigues RP, da Silva VB, Eifler-Lima VL, Kawano DF abio. Identification of a novel putative inhibitor of the Plasmodium falciparum purine nucleoside phosphorylase: exploring the purine salvage pathway to design new antimalarial drugs. Mol Divers. 2017;21: 677–695. 28523625 10.1007/s11030-017-9745-8

[51] Nigatu D, Sobetzko P, Yousef M, Henkel W. Sequence-based information-theoretic features for gene essentiality prediction. BMC Bioinformatics. 2017;18. doi: 10.1186/s12859-017-1884-5 29121868 PMC5679510

[52] Hasibi R, Michoel T, Oyarzún DA. Integration of graph neural networks and genome-scale metabolic models for predicting gene essentiality. Cold Spring Harbor Laboratory; 2023 Aug. doi: 10.1101/2023.08.25.554757 PMC10917767 38448436

