# Supplementary material for: Machine learning methods for predicting essential metabolic genes from Plasmodium falciparum genome-scale metabolic network
Source: PLoS One. 2024 Dec 23;19(12):e0315530. doi: 10.1371/journal.pone.0315530 (PMC11666047; doi:10.1371/journal.pone.0315530)
Supplement: S1 File — This contains a supplementary report on the Mass Flow graph algorithm and a table of the full report of experimental results. (DOCX) [file pone.0315530.s001.docx]

**Supplementary Information: Preliminaries**

**Flux Balance Analysis (FBA)**

FBA is a widely accepted approach to studying cell metabolism and essentiality studies. FBA computes the best steady-state flux distribution of a cell; the flux distribution specifies the cell phenotype [[1–4]](https://www.zotero.org/google-docs/?02pnL9). It accepts a genome scale metabolic model as inputs, containing Stoichiometric matrix as $S$ (i.e, an $n\times m$ matrix containing $n$ metabolite as rows and $m$ reactions in which they are either being produced or consumed as columns). The objective of FBA is to find the solution flux vector **v,** that satisfies the mass balance equation given as $Sv=0$.

Mathematically;

$Max Z=C^{T}V$ $(1)$

Subject to: $\frac{dy}{dt}=SV=0$ and $v_{l}\leq v\leq v_{u}$

where $C$ encodes the cell objective function $v_{l}$ and $v_{u}$ are vectors containing the lower and upper limits on the fluxes of the reactions involved, respectively. Researchers are able to determine flux-flow of the cells under different environmental and genetic conditions by altering the reaction flux bounds [[5,6]](https://www.zotero.org/google-docs/?QfDCKa). FBA has been applied primarily in the studies of gene essentiality prediction via performing single/double gene and/reaction essentiality in silico simulations.

**Mass Flow graphs (MFG)**

MFGs were first proposed by Beguerisse-Díaz *et al.*, [[7]](https://www.zotero.org/google-docs/?zTOcX2)as a way to map flux vectors onto a directed graph that can be analyzed with tools from network science. Each node in these graphs corresponds to a reaction, and two nodes are connected if they both utilize the same metabolite as either reactants or products. One of the best things about these reaction-centered graphs is that you don't have to get rid of pool metabolites like enzyme cofactors, ions, and other molecules that show up in many metabolic reactions which usually manually eliminated in other methods of graph construction to prevent the misleading connections brought about due to the high connectivity they form, tending to dominate and affect the topological structure of the network.

In the MFG construction, these pool metabolites are not removed manually but they tend to map onto weak connections between graph nodes which makes their effectiveness on the overall connectivity much smaller. In order to build a MFG, the weight of the link between reactions $R_{i}$ and $R_{j}$ is defined as the total flow of metabolites generated by $R_{i}$ and consumed by $R_{j}$. Mathematically, an MFG's adjacency matrix may be generated directly from the stoichiometric matrix S and a wildtype FBA solution vector. First, the flow vector is separated into 2 forward and reverse reactions [[1]](https://www.zotero.org/google-docs/?UauSyt):

${V^{*}}_{2m}= \left[ {}_{v^{*-}}^{v^{*+}} \right] = \frac{1}{2} \left[ {}_{abs(v) - v^{*}}^{abs(v) + v} \right]$ (2)

Then redefine the stoichiometric matrix of the GSMM as

$S_{2m}=[S -S] \left[ {}_{0}^{I_{m}}{}_{diag(r)}^{0} \right]$ (3)

Where r, the reversibility vector with $m$ dimensionality representing the reaction’s reversibility as initialize during the GSMN construction, thus:

$r_{j}=\{{}_{0}^{1}{}_{Otherwise}^{If reaction j is reversible}$ (4)

The resulting $S_{2m}$ matrix is used to results in the production and consumption stoichiometric matrices as:

$$Consumption: S_{2m}^{+}=\frac{1}{2}\left( abs\left( S_{2m} \right)+S_{2m} \right)$$

$$Production: S_{2m}^{+}=\frac{1}{2}\left( abs\left( S_{2m} \right)+S_{2m} \right)$$

The flux vector of consumption and production is computed as:

$j_{i}\left( v \right)=S_{2m}^{+}v_{2m}^{*}=S_{2m}^{-}v_{2m}^{*}$ (5)

So, considering a metabolite $X_{ij}$, $j_{i}(v)$ is the flux in which it is produced and consumed. In this case $S_{2m}^{+}$ and $S_{2m}^{-}$ are equal under steady conditions.

Hence the MFG adjacency matrix is computed as:

$M\left( v^{*} \right)=\left( S_{2m}^{+}V^{*} \right)^{T}J_{v}^{\tau}\left( S_{2m}^{-}V^{*} \right)$ (6)

Where;

$$V^{*}=diag(v_{2m}^{*})$$

$$J_{v}=diag\left( j\left( v^{*} \right) \right) and$$

$$\tau=the matrix pseudoinverse of J_{v}$$

**Binary Classification Problem on Mass Flow graphs**

In supervised machine learning, the core objective of binary classification is to find patterns in observations of two classes and use those patterns to automatically classify unseen objects. To evaluate whether a gene is essential or not, the study was focus developing automated classifiers that use growth metrics from knock-out tests and graph features of nodes in the Flux-weighted Reaction Centric Graph.

In this case, we have N pairs of data points, where each pair consists of a binary label y(i) indicating whether the gene is important or not and a p-dimensional feature vector $x(i)$linked to the $i^{th}$ gene/reaction. These feature vectors and labels are arranged into a class label vector (y) and a feature matrix (X) [[8]](https://www.zotero.org/google-docs/?VadpKI).

Mathematically:

$\left[ \left( x^{\left( 1 \right)}, y^{\left( 1 \right)} \right), \left( x^{\left( 2 \right)}, y^{\left( 2 \right)} \right), \left( x^{\left( 3 \right)}, y^{\left( 3 \right)} \right), \ldots, \left( x^{\left( N \right)}, y^{\left( N \right)} \right) \right]$ (7)

where $x^{(i)}\in R^{p}$ is a $p-dimensional$vector features associated with the reaction/genes and $y^{(i)}\in\{0,1\}$ is the class label that is associated with the class label of the reaction or gene. We regard non-essential genes as the negative class (0) and essential genes as the positive class (1) without loss of generality. All the feature vectors are included in the feature matrix, $X$, and the matching class labels are represented by the label vector $y$. The feature vectors and labels are assembled into a feature matrix $X$ and a vector of class labels $y$:

$$X= \left[ x^{\left( 1 \right)}, x^{\left( 2 \right)}, x^{\left( 3 \right)},\ldots, x^{\left( j \right)} \right]$$

$$y= \left[ y^{\left( 1 \right)},y^{\left( 2 \right)}, y^{\left( 3 \right)},\ldots, y^{\left( j \right)} \right]$$

In order to accurately predict labels in the test set, a classification algorithm must first be trained using the input data (train set). Once the system has been trained, it may utilise the feature vectors of future samples for which it has no label information to determine their labels. Assumptions concerning the form of the feature space vary for the various kinds of categorization models. Common models include logistic regression, decision trees, neural networks, and support vector machines. Which model should be utilised depends on the task at hand and the available data. Model training is often accompanied by cross-validation tests in a typical machine learning pipeline to prevent overfitting and ensure the chosen model performs well on untrained datasets.

**Supplementary Table 1: Performance evaluation of ML methods on various feature sets**

| 1. **Adjacency Features** | | | | | | |
| --- | --- | --- | --- | --- | --- | --- |
| ML Model | AuROC | Accuracy | Precision | Recall | F1 - Score | Accuracy 5-Cross Val (5) |
| LogReg | 0.41 | 0.64 | 0.57 | 0.64 | 0.6 | [0.79, 0.79, 0.79, 0.77, 0.77] |
| KNN | 0.48 | 0.74 | 0.59 | 0.74 | 0.66 | [0.68, 0.64, 0.77, 0.77, 0.76] |
| Random Forest | 0.52 | 0.73 | 0.67 | 0.73 | 0.69 | [0.77, 0.74, 0.74, 0.76, 0.76] |
| SVM | 0.5 | 0.77 | 0.6 | 0.77 | 0.67 | [0.79, 0.79, 0.79, 0.77, 0.77] |
| Decision Tree | 0.51 | 0.68 | 0.66 | 0.68 | 0.67 | [0.80, 0.77, 0.62, 0.71, 0.74] |
| **Naive Bayes** | **0.56** | **0.79** | **0.76** | **0.79** | **0.73** | **[0.77, 0.62, 0.79, 0.77, 0.77]** |
| 1. **Topological Features** | | | | | | |
| ML Model | AuROC | Accuracy | Precision | Recall | F1 - Score | Accuracy 5-Cross Val (5) |
| LogReg | 0.46 | 0.71 | 0.59 | 0.71 | 0.64 | [0.79, 0.79, 0.79, 0.77, 0.77] |
| KNN | 0.58 | 0.74 | 0.71 | 0.74 | 0.72 | [0.82, 0.74, 0.73, 0.79, 0.71] |
| **Random Forest** | **0.69** | **0.82** | **0.8** | **0.82** | **0.81** | **[0.85, 0.76, 0.77, 0.74, 0.77]** |
| SVM | 0.5 | 0.77 | 0.6 | 0.77 | 0.67 | [0.79, 0.79, 0.79, 0.77, 0.77] |
| Decision Tree | 0.59 | 0.77 | 0.74 | 0.77 | 0.75 | [0.71, 0.74, 0.73, 0.77, 0.74] |
| Naive Bayes | 0.47 | 0.68 | 0.61 | 0.68 | 0.64 | [0.73, 0.80, 0.79, 0.77, 0.77] |
| 1. **ReFeX** | | | | | | |
| ML Model | AuROC | Accuracy | Precision | Recall | F1 - Score | Accuracy 5-Cross Val (5) |
| LogReg | 0.62 | 0.82 | 0.82 | 0.82 | 0.78 | [0.79, 0.67, 0.76, 0.77, 0.77] |
| KNN | 0.57 | 0.77 | 0.73 | 0.77 | 0.73 | [0.77, 0.74, 0.68, 0.77, 0.77] |
| **Random Forest** | **0.69** | **0.85** | **0.85** | **0.85** | **0.83** | **[0.85, 0.76, 0.74, 0.74, 0.77]** |
| SVM | 0.53 | 0.79 | 0.83 | 0.79 | 0.71 | [0.79, 0.79, 0.79, 0.77, 0.77] |
| Decision Tree | 0.6 | 0.82 | 0.85 | 0.82 | 0.77 | [0.67, 0.76, 0.76, 0.76, 0.79] |
| Naive Bayes | 0.63 | 0.58 | 0.74 | 0.58 | 0.61 | [0.44, 0.58, 0.39, 0.5, 0.56] |

| 1. **RolX** | | | | | | | |  |
| --- | --- | --- | --- | --- | --- | --- | --- | --- |
| ML Model | AuROC | Accuracy | Precision | Recall | F1 - Score | Accuracy 5-Cross Val (5) | | |
| LogReg | 0.5 | 0.77 | 0.6 | 0.77 | 0.67 | [0.79, 0.79, 0.79, 0.77, 0.77] | | |
| KNN | 0.49 | 0.65 | 0.64 | 0.65 | 0.65 | [0.74, 0.82, 0.76, 0.68, 0.59] | | |
| Random Forest | 0.55 | 0.77 | 0.72 | 0.77 | 0.72 | [0.79, 0.76, 0.79, 0.77, 0.74] | | |
| **SVM*** | **0.56** | **0.79** | **0.76** | **0.79** | **73** | **[0.77, 0.79, 0.79, 0.77, 0.77]** | | |
| Decision Tree | 0.49 | 0.76 | 0.56 | 0.76 | 0.67 | [0.74, 0.76, 0.80, 0.77, 0.77] | | |
| Naive Bayes | 0.49 | 0.76 | 0.59 | 0.76 | 0.67 | [0.79, 0.71, 0.80, 0.62, 0.77] | | |
| 1. **ReFeX and RolX** | | | | | | |  |  |
| ML Model | AuROC | Accuracy | Precision | Recall | F1 - Score | Accuracy 5-Cross Val (5) | | |
| LogReg | 0.66 | 0.83 | 0.83 | 0.83 | 0.8 | [0.79, 0.67, 0.76, 0.77, 0.77] | | |
| KNN | 0.59 | 0.8 | 0.79 | 0.8 | 0.76 | [0.77, 0.74, 0.68, 0.77, 0.77] | | |
| **Random Forest** | **0.68** | **0.83** | **0.82** | **0.83** | **0.81** | **[0.83, 0.76, 0.74, 0.74, 0.74]** | | |
| SVM | 0.5 | 0.77 | 0.6 | 0.77 | 0.67 | [0.79, 0.79, 0.79, 0.77, 0.77] | | |
| Decision Tree | 0.62 | 0.82 | 0.82 | 0.82 | 0.78 | [0.74, 0.77, 0.74, 0.77, 0.76] | | |
| Naive Bayes | 0.65 | 0.61 | 0.75 | 0.61 | 0.64 | [0.44, 0.56, 0.39, 0.5, 0.56] | | |
| 1. **TopologyReFeX** | | | | | | |  |  |
| ML Model | AuROC | Accuracy | Precision | Recall | F1 - Score | Accuracy 5-Cross Val (5) | | |
| LogReg | 0.62 | 0.77 | 0.75 | 0.77 | 0.76 | [0.89, 0.74, 0.76, 0.79, 0.76] | | |
| KNN | 0.62 | 0.82 | 0.82 | 0.82 | 0.78 | [0.79, 0.74, 0.68, 0.77, 0.77] | | |
| **Random Forest** | **0.69** | **0.85** | **0.85** | **0.85** | **0.83** | **[0.82, 0.77, 0.74, 0.76, 0.76]** | | |
| SVM | 0.53 | 0.79 | 0.83 | 0.79 | 0.71 | [0.79, 0.79, 0.79, 0.77, 0.77] | | |
| Decision Tree | 0.63 | 0.79 | 0.76 | 0.79 | 0.77 | [0.74, 0.74, 0.73, 0.76, 0.77] | | |
| Naive Bayes | 0.63 | 0.58 | 0.74 | 0.58 | 0.61 | [0.44, 0.62, 0.39, 0.52, 0.56] | | |
| 1. **TopologyRolX** | | | | | | |  |  |
| ML Model | AuROC | Accuracy | Precision | Recall | F1 - Score | Accuracy 5-Cross Val (5) | | |
| LogReg | 0.46 | 0.71 | 0.59 | 0.71 | 0.64 | [0.79, 0.79, 0.79, 0.77, 0.77] | | |
| KNN | 0.66 | 0.8 | 0.78 | 0.8 | 0.79 | [0.82, 0.76, 0.73, 0.79, 0.71] | | |
| **Random Forest** | **0.67** | **0.82** | **0.8** | **0.82** | **0.8** | **[0.82, 0.71, 0.74, 0.73, 0.77]** | | |
| SVM | 0.52 | 0.77 | 0.72 | 0.77 | 0.7 | [0.79, 0.79, 0.79, 0.77, 0.77] | | |
| Decision Tree | 0.61 | 0.8 | 0.78 | 0.8 | 0.77 | [0.76, 0.77, 0.76, 0.77, 0.77] | | |
| Naive Bayes | 0.48 | 0.67 | 0.63 | 0.67 | 0.65 | [0.73, 0.79, 0.73, 0.65, 0.70 ] | | |
| 1. **TopologyReFeXRolX** | | | | | | |  |  |
| ML Model | AuROC | Accuracy | Precision | Recall | F1 - Score | Accuracy 5-Cross Val (5) | | |
| LogReg | 0.61 | 0.76 | 0.74 | 0.76 | 0.74 | [0.89, 0.74, 0.76, 0.79, 0.76] | | |
| KNN | 0.59 | 0.8 | 0.79 | 0.8 | 0.76 | [0.79, 0.74, 0.68, 0.77, 0.77] | | |
| **Random Forest** | **0.66** | **0.83** | **0.83** | **0.83** | **0.8** | **[0.83, 0.72, 0.74, 0.74, 0.77]** | | |
| SVM | 0.53 | 0.79 | 0.83 | 0.79 | 0.71 | [0.79, 0.79, 0.79, 0.77, 0.77] | | |
| Decision Tree | 0.63 | 0.79 | 0.76 | 0.79 | 0.77 | [0.74, 0.77, 0.73, 0.77, 0.77] | | |
| Naive Bayes | 0.65 | 0.61 | 0.75 | 0.61 | 0.64 | [0.42, 0.56, 0.39, 0.5, 0.56] | | |

**Adjacency Features (Table 1A):** We extracted the adjacency matrix from the mass flow graph and used it as 330 nodes by 1010 features matrix traced to the consumption and production stoichiometric matrix (Chapter 3 Equation 3.11). We observed that the ML models had poor predictive power on this dataset. Naïve Bayes achieved the highest performance with auROC of 0.56, accuracy and f1-score, 0.79 and 0.73 respectively, and this is probably due to its ability to work best on high dimensional dataset. Random Forest performed reasonably well with an auROC and accuracy of 0.52 and 0.73 respectively. The accuracy in the 5-fold CV was consistent across all the ML Models.

**Topological Dataset (Table 1B):** We extracted six centrality/topological features from the mass flow graph and used it to train the ML models. We saw that there was improvement in the predictive power across the various ML models compared to adjacency features. Random Forest outperformed other models with an auROC of 0.69 and Accuracy of 0.82. All models achieved relatively improved Accuracy and F1-Score values, indicating good overall performance. The accuracy in 5-fold cross-validation varies slightly across models but generally falls in the range of 0.74 to 0.85.

**ReFeX Feature Set** **(Table 1C):** We extracted 31 feature sets from the mass flow graph using GraphRole. When trained with the ML models, we observed, all models improved performance on this feature sets. Random Forest had the highest AuROC (0.69) and Accuracy (0.85) among all models, indicating its effectiveness with ReFeX features. Logistic Regression and Decision Tree also showed reasonable performance with AuROC values around 0.62 and Accuracy above 0.80. Naive Bayes had a relatively low performance with an AuROC of 0.63 and an Accuracy of 0.58.

**RolX Feature Set (Table 1D):** We generated a set of 5 features based on role clustering of the ReFeX features with GraphRole Algorithm and trained ML models on them. Naive Bayes achieved the highest AuROC (0.56), Accuracy (0.79), and F1-Score (0.73). Random Forest and SVM also showed reasonable performance with AuROC values of 0.55 and 0.56, respectively. The accuracy in 5-fold cross-validation is relatively consistent for all models, with scores mostly around 0.74 to 0.79.

**ReFeX and RolX Feature Set (Table 1E):** We combined ReFeX and RolX features set to form a new dataset which we used for ML training. Random Forest achieved the highest AuROC (0.68) and Accuracy (0.83) among all models, indicating its effectiveness in utilizing the combined features and performing well on the dataset. Logistic Regression, Decision Tree, and KNN also showed reasonable performance with AuROC values around 0.66, 0.62, and 0.59, respectively. Naive Bayes had the lowest Accuracy (0.61) among all models. While it achieved a relatively high Precision (0.75), its Recall (0.61) and F1-Score (0.64) were lower, indicating that it may struggle to correctly identify positive instances.

**TopologyReFeX Feature Set (Table 1F):** We combined Topological and ReFeX feature sets to form a new dataset (TopologyReFeX). Random Forest achieved the highest AuROC (0.69) and Accuracy (0.85) among all models, indicating its strong performance on the dataset with TopologyReFeX features. KNN also performed well with an AuROC of 0.62 and an Accuracy of 0.82, demonstrating its capability to handle the data effectively. Logistic Regression, Decision Tree, and Naive Bayes showed moderate performance, with AuROC values between 0.62 and 0.63 and Accuracies ranging from 0.58 to 0.79. SVM had the lowest AuROC (0.53) and Accuracy (0.79) compared to other models on this dataset.

**TopologyRolX Feature Set (Table 1G):** We combined Topological and RolX datasets to form a new dataset as TopologyRolX. KNN achieved the highest AuROC (0.66) and Accuracy (0.80) among all models, indicating its strong performance on the dataset with TopologyRolX features. Random Forest and Decision Tree also showed competitive performance with AuROC values around 0.67 and Accuracies of 0.82. Logistic Regression, SVM, and Naive Bayes had relatively lower performance, with AuROC values between 0.46 and 0.52 and Accuracies ranging from 0.67 to 0.77.

**TopologyReFeXRolX Dataset (Table 1H):** We finally combined the three-feature set, Topological, ReFeX and RolX features to form a new dataset. Random Forest achieved the highest AuROC (0.66) and Accuracy (0.83) among all models, indicating its strong performance on the dataset with TopologyReFexRolX features. Logistic Regression, Decision Tree, and KNN also showed competitive performance with AuROC values around 0.61 to 0.63 and Accuracies ranging from 0.76 to 0.80. Naive Bayes had a higher AuROC (0.65) compared to other datasets, but it showed the lowest Accuracy (0.61) and F1-Score (0.64). SVM had the lowest AuROC (0.53) and performed relatively lower in terms of Accuracy (0.79) and F1-Score (0.71) on this dataset.

**Supplementary Table 2: List of False Positive Prediction (Genes Labeled as non-essential but predicted as essential)**

| **Reaction** | **Gene** | **Binary labels** | **ML Prediction** |
| --- | --- | --- | --- |
| ACONTb | PF3D7_1342100 | NE | E |
| MAN6PI | PF3D7_0801800 | NE | E |
| PPPGO6m | PF3D7_1028100 | NE | E |
| PYNP2r | PF3D7_0513300 | NE | E |
| TMPPP | PF3D7_0614000 | NE | E |
| THBPT4ACAMDASE | PF3D7_1108300 | NE | E |
| CITtcm | PF3D7_1223800 | NE | E |
| DHORTS | PF3D7_1472900 | NE | E |
| SUCOAS1m | (PF3D7_1437700 or PF3D7_1431600) and PF3D7_1108500 | NE | E |

**References**

[1. Freischem LJ, Barahona M, Oyarzún DA. Prediction of gene essentiality using machine learning and genome-scale metabolic models. Cold Spring Harbor Laboratory; 2022 Mar. doi:10.1101/2022.03.31.486520](https://www.zotero.org/google-docs/?D81Uxv)

[2. Gatto F, Miess H, Schulze A, Nielsen J. Flux balance analysis predicts essential genes in clear cell renal cell carcinoma metabolism. Sci Rep. 2015;5. doi:10.1038/srep10738](https://www.zotero.org/google-docs/?D81Uxv)

[3. Sahu A, Blätke M-A, Szymański JJ, Töpfer N. Advances in flux balance analysis by integrating machine learning and mechanism-based models. Comput Struct Biotechnol J. 2021;19: 4626–4640. doi:10.1016/j.csbj.2021.08.004](https://www.zotero.org/google-docs/?D81Uxv)

[4. Wu SG, Wang Y, Jiang W, Oyetunde T, Yao R, Zhang X, et al. Rapid prediction of bacterial heterotrophic fluxomics using machine learning and constraint programming. PLOS Comput Biol. 2016;12: e1004838. doi:10.1371/journal.pcbi.1004838](https://www.zotero.org/google-docs/?D81Uxv)

[5. Martins Conde P do R, Sauter T, Pfau T. Constraint based modeling going multicellular. Front Mol Biosci. 2016;3. doi:10.3389/fmolb.2016.00003](https://www.zotero.org/google-docs/?D81Uxv)

[6. Yasemi M, Jolicoeur M. Modelling Cell Metabolism: A Review on Constraint-Based Steady-State and Kinetic Approaches. Processes. 2021;9: 322. doi:10.3390/pr9020322](https://www.zotero.org/google-docs/?D81Uxv)

[7. Beguerisse-Díaz M, Bosque G, Oyarzún D, Picó J, Barahona M. Flux-dependent graphs for metabolic networks. Npj Syst Biol Appl. 2018;4. doi:10.1038/s41540-018-0067-y](https://www.zotero.org/google-docs/?D81Uxv)

[8. Vijayakumar S, Rahman PKSM, Angione C. A hybrid flux balance analysis and machine learning pipeline elucidates metabolic adaptation in cyanobacteria. iScience. 2020;23: 101818. doi:10.1016/j.isci.2020.101818](https://www.zotero.org/google-docs/?D81Uxv)
